# Perceived Exertion Is Associated with Cardiovascular Strain but Not Glycemic Response to Gym-Based Exercise in Adults with Type 1 Diabetes: An Exploratory Randomized Crossover Trial

**DOI:** 10.3390/ijerph23060814

**Published:** 2026-06-19

**Authors:** José Adevalton Feitosa Gomes, Anthony Rodrigues de Vasconcelos, José Roberto Andrade do Nascimento Júnior, Ysadora Verena Ribeiro de Souza, Fabiana Oliveira dos Santos Camatari, Bruno Bavaresco Gambassi, Manoel da Cunha Costa, Paulo Adriano Schwingel, Jorge Luiz de Brito Gomes

**Affiliations:** 1 Programa de Pós-Graduação em Reabilitação e Desempenho Funcional (PPGRDF), Universidade de Pernambuco (UPE), Petrolina 56328-900, PE, Brazil; adevaltonfeitosa@hotmail.com (J.A.F.G.); anthony.vasconcelos@upe.br (A.R.d.V.); manoel.costa@upe.br (M.d.C.C.); jorge.brito@univasf.edu.br (J.L.d.B.G.); 2 Laboratório de Pesquisas em Desempenho Humano (LAPEDH), Universidade de Pernambuco (UPE), Petrolina 56328-900, PE, Brazil; fabiana.camatari@upe.br (F.O.d.S.C.); professorbrunobavaresco@gmail.com (B.B.G.); 3 Colegiado de Educação Física (CEFIS), Universidade Federal do Vale do São Francisco (UNIVASF), Petrolina 56304-917, PE, Brazil; joseroberto.nascimentojunior@univasf.edu.br (J.R.A.d.N.J.); ysadora.verena@discente.univasf.edu.br (Y.V.R.d.S.); 4 Programa de Pós-Graduação em Gestão e Atenção à Saúde (PPGGAS), Universidade Ceuma (UNICEUMA), São Luís 65075-120, MA, Brazil; 5 Escola Superior de Educação Física (ESEF), Universidade de Pernambuco (UPE), Recife 50100-130, PE, Brazil

**Keywords:** type 1 diabetes, perceived exertion, enjoyment, cardiovascular strain, physical exercise, blood glucose, cardiovascular risk

## Abstract

**Highlights:**

**Public health relevance—How does this work relate to a public health issue?**

- Adults with type 1 diabetes mellitus (T1DM) carry an elevated cardiovascular risk profile, and regular exercise is a cornerstone non-pharmacological strategy to mitigate this risk.
- In real-world gym settings, however, continuous cardiovascular and glycemic monitoring is rarely available, limiting the safe and effective implementation of exercise prescriptions in this at-risk population.

**Public health significance—Why is this work of significance to public health?**

- This exploratory randomized crossover trial suggests that ratings of perceived exertion (RPE), but not enjoyment, may provide information about relative cardiovascular strain during gym-based aerobic and resistance training in adults with T1DM when exercise session type is considered.
- Conversely, neither RPE nor enjoyment was associated with acute capillary blood glucose responses, indicating that direct glycemic monitoring remains essential for exercise safety in this population.

**Public health implications—What are the key implications or messages for practitioners, policy makers and/or researchers in public health?**

- Exercise professionals may consider using post-session RPE as an accessible adjunct to inform cardiovascular strain monitoring in adults with T1DM when sophisticated monitoring tools are unavailable, supporting more equitable access to safe exercise prescriptions. Enjoyment, while potentially relevant for exercise adherence, was not independently associated with cardiovascular strain in this exploratory study.
- Replication in larger samples is needed before these exploratory findings can inform clinical guidelines or public health recommendations.

**Abstract:**

Adults with type 1 diabetes mellitus (T1DM) face elevated cardiovascular risk, and regular exercise is a key non-pharmacological mitigation strategy. However, safe prescription requires cardiovascular and glycemic monitoring, often unfeasible in real-world gyms. Low-cost psychophysiological tools (ratings of perceived exertion—RPE and enjoyment) may offer practical alternatives. This exploratory randomized crossover trial examined whether post-session RPE and enjoyment are associated with acute heart rate (HR) and capillary blood glucose (BG) responses to gym-based aerobic and resistance training. Twelve adults with T1DM (29.8 ± 7.8 years; HbA1c 7.7 ± 1.6%; LDL-c 119.5 ± 24.4 mg/dL) completed three ~30 min sessions: aerobic interval training (AE) and two resistance protocols (STA, STB). HR and BG were measured pre-, immediately post-, and 20 min post-exercise; RPE and enjoyment, post-session. Multiple linear regression, controlling for exercise session type, examined associations of RPE and enjoyment with resting HR, BG, and percentage of heart rate reserve (%HR). RPE was higher after STA and STB than AE (*p* < 0.001; *η*^2^*p* = 0.529), while enjoyment and %HR were similar across sessions. Neither variable was associated with resting HR or BG (all adjusted *R*^2^ < 0; all *p* > 0.05). Controlling for exercise session type, RPE was a significant positive predictor of %HR (*β* = 0.44, *p* = 0.044), whereas enjoyment was not (*β* = −0.06, *p* = 0.719); however, the overall %HR model did not reach statistical significance (adjusted *R*^2^ =0.119; *F*(4,31) = 2.183; *p* = 0.094). These exploratory findings suggest that RPE, but not enjoyment, may serve as a low-cost adjunct intensity marker to inform exercise prescription in adults with T1DM at elevated cardiovascular risk; however, replication in larger samples is needed before clinical recommendations can be drawn. Direct BG monitoring remains essential for safety.

## 1. Introduction

Exercise represents a cornerstone non-pharmacological strategy for health optimization and glycemic management in individuals with type 1 diabetes mellitus (T1DM). However, acute glucose responses to exercise exhibit marked interindividual variability depending on exercise modality and intensity. Monitoring physiological responses, including blood glucose (BG) and heart rate (HR), before and after exercise is therefore critical to minimize hypoglycemic and hyperglycemic episodes during routine training. Moreover, different exercise formats elicit distinct counterregulatory and metabolic responses, explaining why comparable gym sessions can produce divergent cardiovascular and glycemic patterns in individuals with T1DM [1,2,3].

Despite well-established benefits, numerous barriers impede regular exercise participation in T1DM populations. Chief among these are the fear of exercise-induced hypoglycemia and the daily complexity of adjusting insulin and carbohydrate intake around workouts [4,5]. Qualitative evidence reveals that perceived barriers and motivators (including safety concerns, confidence, social context, and enjoyment) shape exercise adherence in adults with T1DM. Participation is not solely physiologically determined [6]. In practical gym settings, this behavioral and logistical burden intensifies when continuous monitoring devices are unavailable, creating demand for rapid, cost-effective tools that guide session intensity while ensuring glycemic safety [7].

When continuous monitoring is limited, low-cost psychophysiological measures offer practical alternatives. These measures translate exercise guidance into routine gym practice, particularly by capturing internal load and affective experience [7]. Ratings of Perceived Exertion—RPE (typically assessed using Borg-type scales [8])—reflect integrated effort perception and are widely employed to monitor and prescribe exercise intensity. The OMNI-RES scale specifically quantifies perceived exertion during resistance training, supporting intensity control in gym-based programs [9]. Strong correlations between RPE and relative cardiovascular strain (e.g., %HRmax) during steady-state exercise establish RPE as a viable surrogate marker of intensity when laboratory measures are inaccessible [10]. Similarly, enjoyment (typically assessed via simple visual analog scales) has been associated with exercise engagement and can be rapidly evaluated post-exercise, including in T1DM populations [11,12].

Nevertheless, whether post-session RPE and enjoyment are associated with acute HR and BG responses following typical gym-based aerobic and resistance sessions in adults with T1DM remains unclear. The substantial variability in acute physiological responses to exercise in T1DM, coupled with practical monitoring barriers, underscores the need for simple, field-applicable markers to guide exercise prescription and safety in routine gym settings [2].

Although RPE and enjoyment are feasible measures that have characterized exercise responses in T1DM, evidence remains limited regarding their potential associations with acute cardiovascular and glycemic responses across standard aerobic and resistance gym sessions [11,12]. Therefore, this exploratory randomized crossover trial aimed to examine whether post-session RPE and enjoyment are associated with acute physiological responses (HR-derived intensity and capillary BG changes) following aerobic and strength training sessions in adults with T1DM. We hypothesized that RPE and enjoyment would be associated with relative cardiovascular strain (%HRmax) across exercise modalities. We further explored whether these psychophysiological measures would similarly track acute capillary blood glucose responses, given the multifactorial nature of glycemic regulation in T1DM.

## 2. Materials and Methods

### 2.1. Study Design and Ethical Approval

This randomized crossover trial examined three exercise conditions (aerobic, strength session A, and strength session B) in adults with T1DM. The trial was registered in the Brazilian Registry of Clinical Trials (RBR-57T7VB; UTN: U1111-1237-5864) and approved by the Research Ethics Committee at the Federal University of Vale do São Francisco (UNIVASF) with Report Number 3.349.261 and Certificate of Presentation for Ethical Appreciation (CAAE) Number 01481718.9.0000.5196. All participants provided written informed consent, and procedures followed the Declaration of Helsinki. In addition, this study was reported in accordance with the Consolidated Standards of Reporting Trials (CONSORT) 2025 guidelines.

### 2.2. Participants and Recruitment

Participants were recruited through social media advertisements and flyers distributed via the extension program ‘*Exercício Físico como Açúcar Diário*’ [‘Exercise as Daily Sugar’], offered by the Colegiado de Educação Física (CEFIS) at UNIVASF (Petrolina, Pernambuco, Brazil). No prospective sample-size calculation was performed. A post hoc power analysis was conducted after data collection solely to characterize effect detectability, assuming *α* = 0.05 and power (1 − *β*) ≥ 0.80 to detect the smallest anticipated psychological effect size (enjoyment), using G*Power software (Heinrich-Heine-Universität Düsseldorf, Düsseldorf, Germany, release 3.1.9.4, 2019). This analysis indicated that a minimum of 12 participants would have been necessary to detect the target effect under the assumed conditions [13]. Recruitment was conducted between June 2019 and August 2019, and exercise sessions were performed between August 2019 and February 2020.

### 2.3. Inclusion and Exclusion Criteria

Eligible participants were adults with T1DM aged 18–45 years, of both sexes, without medical contraindications to exercise or secondary complications that could be exacerbated by study participation (e.g., peripheral or central neuropathy, advanced retinopathy, vascular disease, or amputations, as diagnosed by their physician). Exclusion criteria comprised: failure to complete all three exercise sessions; initiation of a new structured exercise program during the study period; occurrence of musculoskeletal injury preventing training session performance; or withdrawal at the request of the treating physician.

### 2.4. Experimental Design and Randomization

Following baseline assessments (anthropometry, body composition, and fasting blood sampling) on day 1, participants waited 48 h before attending a second visit (day 2), during which they familiarized themselves with resistance exercises and completed a 12 min field test to estimate maximal oxygen uptake (VO_2_max). Subsequently, each participant completed three experimental sessions—one aerobic (AE) and two strength sessions (STA and STB)—in randomized crossover order, with 48–196 h between sessions scheduled on separate days (days 3, 4, and 5). These intervals were designed to minimize residual fatigue and carryover effects while enabling flexible scheduling for participants.

After the preliminary assessments were completed, participants were assigned to one of three possible sequences of crossover exercise sessions. Each sequence had an equal number of participants in a 1:1:1 ratio as follows: sequence i, STA followed by STB and then AE; sequence ii, STB followed by AE and then STA; or sequence iii, AE followed by STA and then STB. The randomization process was performed using a computer-generated random sequence, while allocation to the respective groups was concealed within sequentially numbered, opaque, sealed envelopes. These envelopes were prepared by a researcher who was not involved in recruitment or data collection.

Due to the nature of the exercise interventions, blinding of participants and personnel delivering the sessions was not feasible; the statistical analyst was not blinded to session allocation.

### 2.5. Protocols and Instruments

#### 2.5.1. Anthropometry and Body Composition Measures

Total body mass (kilograms—kg) and height (centimeters—cm) were measured using a calibrated combined LD-1050 stadiometer and digital scale (Lider Balanças, Araçatuba, SP, Brazil). Body mass index (BMI) was calculated as total body mass (kg) divided by height squared (m^2^). Body circumferences were assessed using a TR 4010 anthropometric tape (Sanny, São Bernardo do Campo, SP, Brazil), and skinfold thicknesses were measured with a clinical skinfold caliper (Sanny), following standard anthropometric procedures.

#### 2.5.2. Blood Sample

Venous blood samples were obtained from the antecubital vein by a certified phlebotomist in a clinical laboratory. Glycated hemoglobin (HbA1c), high-density lipoprotein (HDL) cholesterol, low-density lipoprotein (LDL) cholesterol, very-low-density lipoprotein (VLDL) cholesterol, total cholesterol, triglycerides, hematocrit and creatinine were analyzed using standard automated methods according to the laboratory’s routine procedures.

#### 2.5.3. Estimation of Training Loads

The Brzycki equation was used to estimate one-repetition maximum (1RM) values for resistance training [14], from which 60% of 1RM was prescribed for each exercise. For the aerobic condition, running speeds corresponding to 40% and 60% of estimated VO_2_max were calculated from the 12 min field test completed on day 2, following previously described procedures in T1DM populations [12].

For the STA session, submaximal tests to voluntary fatigue estimated 1RM for seven exercises: bench press, horizontal leg press, biceps curl, leg extension, machine shoulder press, hip adductor machine, and abdominal crunch. Loads corresponding to 60% of 1RM were prescribed. For STB, the same procedure was applied to the following exercises: lat pulldown, seated leg curl, triceps pulley, seated row, trapezius pull, hip abductor machine, and isometric plank.

#### 2.5.4. Session Procedures and Timing

Before each experimental session, participants rested seated for 10 min while resting HR, blood pressure, and capillary blood glucose were measured. Following this rest period, they performed the assigned training session (AE, STA, or STB), which lasted approximately 30 min.

Immediately post-session, participants returned to the seated position for repeat measurements of HR, blood pressure, and blood glucose, and for assessment of ratings of perceived exertion (RPE; Borg 6–20 scale for AE, OMNI-RES 0–10 scale for STA and STB) [15,16] and enjoyment. Twenty minutes after exercise, HR, systolic and diastolic blood pressure, and capillary blood glucose were measured again, with participants seated and instructed to remain silent and motionless during measurements.

All sessions were scheduled between 16:00 and 19:00 h. Following current diabetes exercise recommendations, participants were instructed to reduce rapid-acting insulin at the last pre-session meal by 50% to mitigate exercise-induced hypoglycemia risk [1]. Adverse events (e.g., severe hypoglycemia, hypotension, musculoskeletal injury) were monitored throughout each session by the supervising researcher and registered in the participant’s logbook. Capillary blood glucose was checked immediately before each session; participants presenting with pre-exercise BG below 100 mg·dL^−1^ or above 350 mg·dL^−1^ were excluded from that session following current safety recommendations [1]. Fast-acting carbohydrate supplementation (15–20 g) was available throughout all sessions to manage hypoglycemic episodes if required.

#### 2.5.5. Physiological Responses (Hemodynamic and Capillary Blood Glucose)

HR (beats per minute—bpm) was recorded using an FT1 heart rate monitor (Polar Electro Oy, Kempele, Finland). Hemodynamic measurements were obtained in the seated position at three time points: pre-exercise, immediately post-exercise, and 20 min post-exercise. At each time point, participants remained seated, silent, and motionless during measurements. Mean HR values for each time point and session were analyzed, and relative exercise intensity was expressed as a percentage of heart rate reserve (%HR) following procedures described in previous T1DM studies [12,17]. %HR was calculated as: %HR = [(exercise HR − resting HR)/(age-predicted maximum HR − resting HR)] × 100, where age-predicted maximum HR was estimated as 220 − age (years).

Capillary blood glucose (BG, mg·dL^−1^) was assessed from fingertip samples using a portable glucometer Accu-Chek Active (Roche Diagnostics GmbH, Mannheim, Germany), an Accu-Chek Softclix spring-loaded lancing device, disposable test strips, and disposable lancets (Roche Diagnostics GmbH), following manufacturer instructions and previously published T1DM protocols [11,12]. Blood glucose was measured at the same three time points as hemodynamic variables, with participants seated and instructed not to speak or move.

#### 2.5.6. Psychophysiological Scales

In the AE session, RPE was self-reported using the 6–20 Borg scale [8]. To facilitate comparison with strength sessions, RPE values were converted to a 0–10 metric for statistical analyses, following previously described procedures [18]. RPE was defined to participants as: “How tired did your body feel while you were exercising, considering your breathing and working muscles?”

In STA and STB sessions, RPE was assessed using the OMNI-RES 0–10 scale, developed to quantify effort during resistance exercise [16]. Participants were asked: “How tired did your body feel while exercising, considering the exertion and discomfort in your working muscles?” The use of RPE as an intensity metric is supported by robust correlations between perceived exertion and HR, demonstrated in earlier studies [19].

Enjoyment was measured immediately after RPE assessment using a 100 mm visual analog scale adapted from previous work [11,12,20]. The paper-based scale ranged from 0 (“very boring”) to 100 (“very enjoyable”), and participants marked a point reflecting how much they enjoyed the session as a whole. This mood analog scale has demonstrated good test–retest reliability (r ≈ 0.89). Raw scores (in mm) were subsequently divided by 10 for statistical analyses and reporting, yielding a 0–10 scale to facilitate direct comparison with RPE values; regression coefficients are therefore expressed per unit of this 0–10 scale.

#### 2.5.7. Aerobic and Muscular Strength Training Sessions (AE, STA, and STB)

The AE session consisted of interval training, alternating 1 min at the speed corresponding to 40% of estimated VO_2_max with 1 min at 60% of estimated VO_2_max, following a 2 min warm-up at 40% of estimated VO_2_max. Total AE session duration was approximately 30 min.

Strength training sessions (STA and STB) were performed at moderate intensity using loads corresponding to 60% of estimated 1RM, with a cadence of approximately 3 s per repetition (1.5 s eccentric, 1.5 s concentric) and inter-set intervals of 50–60 s. Before each strength session, participants completed one warm-up set at 50% of their estimated 1RM for each exercise, followed by a 60 s interval before the main sets, performed over approximately 30 min.

### 2.6. Statistical Analysis

Data are presented as mean ± standard deviation (SD) or standard error (SE), as indicated. Physiological and psychophysiological variables across the three sessions (AE, STA, STB) and time points (pre, immediately post, 20 min post) were compared using one-way repeated-measures analysis of variance (ANOVA) with Bonferroni post hoc tests, treating session as a within-subject factor. Effect sizes (partial *η*^2^: *η*^2^*p*) were calculated to quantify the magnitude of between-session differences and interpreted as small (*η*^2^*p* = 0.01), medium (*η*^2^*p* = 0.06), or large (*η*^2^*p* = 0.14), according to Cohen’s recommendations [21]. Sphericity was assessed using Mauchly’s test; where the assumption was violated, Greenhouse–Geisser-corrected degrees of freedom and *p*-values are reported.

Multiple linear regression analyses examined associations between psychophysiological variables (RPE and enjoyment) and physiological outcomes (resting HR, BG at each time point, and %HR). Given the crossover design, exercise session type (AE, STA, and STB; reference category: STB) was included as a covariate in all models using two binary dummy variables, to control for modality-specific differences in cardiovascular and metabolic demands. Analyses were conducted on the pooled dataset comprising 36 observations (12 participants × 3 sessions). It is acknowledged that this approach does not fully account for within-participant clustering inherent to the crossover design; accordingly, findings should be interpreted as exploratory and not as confirmatory, and no inference of independence across observations is implied. Before model fitting, multicollinearity was assessed using variance inflation factors (VIF), which ranged from 1.10 to 2.37—below the conventional threshold of 5–10 considered acceptable [22].

All statistical analyses were conducted using IBM SPSS Statistics (IBM Corp., Armonk, NY, USA, release 25.0, 2017). A two-sided *p*-value ≤ 0.05 was considered statistically significant.

## 3. Results

**Figure 1:**
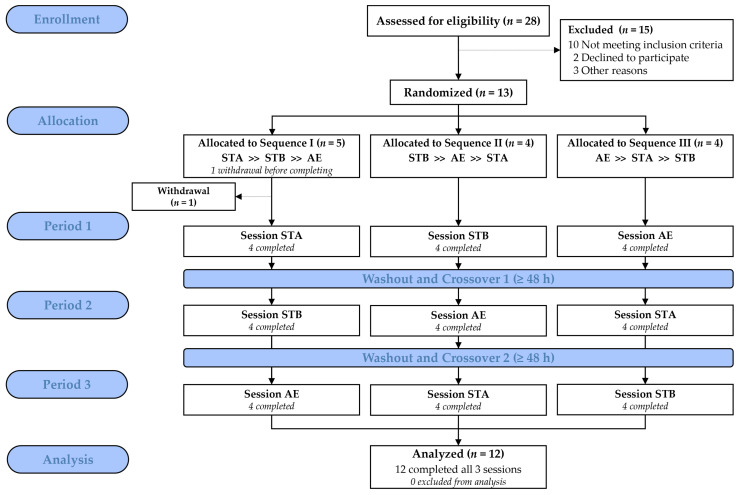
Study design and participant flow (CONSORT). Of 28 adults assessed for eligibility, 13 were randomized across three sequence orders (1:1:1 ratio). All 12 participants who completed the protocol were allocated to each of the three exercise conditions (AE, STA, STB) in a crossover fashion and are included in the final analysis.

The study design is illustrated in Figure 1 (CONSORT flow diagram). Of 28 adults with T1DM assessed for eligibility, 13 were randomized to the exercise protocol, and 12 completed all sessions and were included in the final analyses.

Baseline anthropometric, clinical, and laboratory characteristics of the 12 participants are presented in Table 1. The sample comprised young to middle-aged adults (mean age 29.8 ± 7.8 years; 7 males, 5 females) with a mean T1DM duration of 11.9 ± 5.2 years. Participants exhibited suboptimal glycemic control (HbA1c 7.7 ± 1.6%), normal body mass index (25.2 ± 4.6 kg·m^−2^), and low-density lipoprotein (LDL) cholesterol of 119.5 ± 24.4 mg·dL^−1^. This LDL concentration exceeds the guideline-recommended target of <100 mg·dL^−1^ for individuals with T1DM and elevated cardiovascular risk.

Exploratory analyses of primary psychophysiological outcomes revealed no significant sex differences in RPE after AE (male: 4.4 ± 1.8 vs. female: 3.4 ± 1.2, *p* = 0.298), STA (male: 5.9 ± 1.2 vs. female: 6.2 ± 1.0, *p* = 0.638), or STB (male: 6.7 ± 1.2 vs. female: 6.6 ± 1.0, *p* = 0.873). Similarly, enjoyment scores did not differ significantly between sexes after AE (male: 4.7 ± 2.2 vs. female: 7.3 ± 2.0, *p* = 0.089), STA (male: 5.9 ± 2.2 vs. female: 8.1 ± 1.4, *p* = 0.075), or STB (male: 7.3 ± 1.7 vs. female: 7.5 ± 1.3, *p* = 0.808). Given the small and unequal sex distribution (7 males, 5 females), subsequent analyses pragmatically pooled all participants. The absence of statistically significant sex differences does not indicate equivalence in this underpowered subgroup comparison and should not be interpreted as such.

Physiological and psychophysiological responses for each session (AE, STA, STB) are presented in Table 2. Mean capillary BG decreased from pre- to immediately post-exercise in all three sessions: −45.3 mg·dL^−1^ (AE), −37.0 mg·dL^−1^ (STA), and −37.8 mg·dL^−1^ (STB). Mean changes from pre- to 20 min post-exercise were −46.1 mg·dL^−1^ (AE), −45.2 mg·dL^−1^ (STA), and −29.6 mg·dL^−1^ (STB), indicating that all three modalities elicited a reduction in glycemia. While AE and STA maintained or further reduced BG at 20 min post-exercise, STB showed a partial glycemic recovery relative to the immediately post-exercise value. Resting HR, pre-exercise BG, BG immediately post-exercise, and BG at 20 min post-exercise did not differ significantly between sessions (all *p* > 0.05). Conversely, RPE differed significantly between sessions (*F*(2,22) = 12.368, *p* < 0.001; *η*^2^*p* = 0.529). Higher RPE values were observed following STA and STB compared with AE, indicating greater perceived exertion during resistance training sessions. No statistically significant between-session differences were observed for enjoyment or %HR (all *p* > 0.05).

Multiple linear regression analyses examining associations between RPE and enjoyment with physiological variables, controlling for exercise session type, are summarized in Table 3. Models incorporating RPE, enjoyment, and session type (aerobic, strength A, and strength B; reference: strength B) as a covariate did not account for meaningful variance in resting HR or BG at any time point (pre-exercise, immediately post-exercise, or 20 min post-exercise). Adjusted *R*^2^ values ranged from −0.094 to −0.025 (all *p* > 0.05). For %HR, the overall model did not reach statistical significance (adjusted *R*^2^ = 0.119; *F*(4,31) = 2.183; *p* = 0.094). Within this model, however, RPE was a significant positive predictor (*B* = 3.05, 95% confidence interval [CI]: 0.08 to 6.02; *p* = 0.044). This indicated a meaningful partial association with relative cardiovascular strain when exercise session type and enjoyment were controlled. Enjoyment was not a significant predictor of %HR (*B* = −0.33, 95%CI: −2.18 to 1.52; *p* = 0.719) nor of any other physiological outcome. Neither RPE nor enjoyment was associated with acute BG changes across modalities. Finally, no adverse events were observed during the trial.

## 4. Discussion

This randomized crossover trial examined whether psychophysiological variables (RPE and enjoyment) were associated with acute physiological responses following aerobic and resistance training sessions in adults with T1DM. The primary findings revealed that RPE and enjoyment showed no significant associations with capillary blood glucose responses across exercise modalities. However, when controlling for exercise session type, RPE was a significant positive predictor of %HR (*B* = 3.05; *β* = 0.44; *p* = 0.044), whereas enjoyment was not significantly associated with any physiological outcome (all *p* > 0.05).

### 4.1. Blood Glucose Responses

Contrary to our hypothesis, neither RPE nor enjoyment were associated with acute capillary blood glucose changes following AE, STA, or STB. This contrasts with prior research demonstrating strong correlations between psychophysiological responses and physiological strain across various exercise contexts, including high-intensity training and competitive settings [23,24,25].

One plausible explanation is the duration- and intensity-matched design of the present study. All sessions were performed at moderate intensity for approximately 30 min, which may have minimized between-session variability in glycemic responses. As demonstrated previously [11,12], glucose regulation during exercise in T1DM is multifactorial. Under more demanding conditions—such as higher or maximal intensity, longer duration, or less standardized insulin and carbohydrate strategies—greater divergence in BG responses might emerge, particularly among individuals sustaining higher workloads. This divergence could relate to stronger counterregulatory hormonal activity, especially when carbohydrate strategies are less standardized [26].

Evidence from adolescents with insulin-dependent diabetes mellitus (IDDM) indicates that RPE during prolonged cycling is influenced by both metabolic support and training status. RPE was 15–25% higher in adolescents with IDDM compared with controls during 60 min at 60% of peak oxygen uptake, and glucose ingestion attenuated RPE in the IDDM group [27]. Conversely, well-trained adolescents with IDDM exhibited RPE responses comparable to healthy controls during moderate-intensity cycling, suggesting that training status and carbohydrate availability influence perceived exertion more substantially than diabetes per se [26]. These findings, combined with our predominantly homogeneous and moderately active sample, may explain the absence of association between psychophysiological measures and BG prediction.

### 4.2. Heart Rate Responses

Partially supporting our hypothesis, RPE was a significant positive predictor of %HR within the regression model (*B* = 3.05; *β* = 0.44; *p* = 0.044). However, the overall model did not reach statistical significance (adjusted *R*^2^ = 0.119; *F*(4,31) = 2.183; *p* = 0.094). Enjoyment was not a significant predictor of %HR (*B* = −0.33; *p* = 0.719), indicating that perceived exertion, but not enjoyment, was associated with relative cardiovascular strain during sessions.

It should be noted that the overall model’s non-significance does not negate RPE’s partial association with %HR. The two session-type dummy covariates absorbed degrees of freedom without proportionally increasing explained variance, attenuating the global *F*-statistic while preserving RPE’s contribution. This pattern is consistent with the exploratory and underpowered nature of this analysis. Given the small sample size and the overall non-significance of the %HR model, this partial association should be regarded as preliminary and hypothesis-generating only.

This pattern aligns with the broader literature showing that habitual physical activity level and affective responses influence both physiological reactivity and perceived strain. Individuals with T1DM often avoid exercise due to fear of hypoglycemia, which may limit conditioning and maintain elevated RPE for a given workload [28,29]. Lower training status and less positive affect are associated with higher perceived exertion and poorer long-term adherence [2,28,30]. Our results suggest that, even under standardized moderate-intensity conditions, RPE provides meaningful information about cardiovascular strain in this population, whereas enjoyment was not statistically significantly associated with cardiovascular strain [26,31,32,33]. Collectively, these findings suggest that although enjoyment may not strongly predict acute cardiovascular strain, monitoring both RPE and enjoyment may inform exercise prescription tolerability and long-term sustainability in adults with T1DM.

The relevance of enjoyment as a behavioral construct is supported by qualitative evidence identifying it as a key motivator for exercise adherence in adults with T1DM, alongside perceived physical benefits, body image, and social interaction [6]. In healthy individuals, higher-intensity protocols can elicit equal or greater enjoyment compared with moderate continuous exercise, particularly when performed as interval training [26,31,32,33].

### 4.3. Limitations and Strengths

Several limitations warrant consideration. First, the small single-center sample (*n* = 12 participants, with 36 pooled observations used in regression analyses) limits generalizability and precludes strong causal inferences despite the randomized crossover design. Second, only one intensity domain (moderate) and one session duration (~30 min) were tested; different results might emerge at higher intensities, longer durations, or with alternative exercise modalities. Third, although exercise session type was included as a covariate in all regression models to account for the crossover design, the small number of pooled observations (*n* = 36) limits statistical power and may have reduced the likelihood of detecting modest associations between psychophysiological and physiological variables. Fourth, the limited variability in enjoyment scores across sessions (means ranging from 5.8 to 7.4 on a 0–10 scale; *p* = 0.216) may have reduced statistical power to detect associations between enjoyment and acute physiological outcomes. Fifth, order and carryover effects were not formally tested; the balanced 1:1:1 sequence allocation and a minimum 48 h washout period were designed to minimize these effects, although the broad range of inter-session intervals (48–196 h) introduced variability in participants’ baseline metabolic state that could not be controlled post hoc. Sixth, individual meal composition, meal-to-exercise timing, insulin type and regimen, and time since last bolus were not systematically recorded, which may have contributed to inter-individual variability in blood glucose responses. Seventh, the linear transformation of Borg 6–20 values to a 0–10 metric, while validated [18], introduces uncertainty regarding full psychometric comparability with the OMNI-RES scale across modalities. Finally, mild hypoglycemic episodes requiring self-administered carbohydrate correction were not systematically recorded; future studies should prospectively document all glycemic events, including self-treated hypoglycemia.

Conversely, this study has notable strengths, including the randomized crossover design, use of real-world gym protocols (AE, STA, STB) with standardized insulin adjustments, and combined analysis of RPE, enjoyment, HR, and BG. These features enhance ecological validity for practitioners supervising exercise in adults with T1DM.

### 4.4. Practical Implications

From a practical standpoint, monitoring RPE after gym-based aerobic and resistance training sessions may help clinicians and exercise professionals adjust intensity and volume to achieve appropriate cardiovascular strain. Monitoring enjoyment may inform strategies to preserve positive affective experience and support long-term adherence. Given that these tools are easy to administer, low-cost, and quick to learn, they represent feasible adjuncts to HR and BG monitoring in T1DM populations. However, practitioners should recognize that while RPE demonstrated a meaningful association with cardiovascular strain (*β* = 0.44), enjoyment contributed minimally to acute physiological prediction despite its importance for behavioral outcomes.

## 5. Conclusions

This randomized crossover study demonstrated that psychophysiological variables were significantly associated with relative HR responses, but not with capillary blood glucose following gym-based aerobic and resistance training sessions in adults with T1DM. Specifically, RPE was a significant positive predictor of %HR within the regression model (*B* = 3.05; *β* = 0.44; *p* = 0.044), although the overall model did not reach statistical significance (adjusted *R*^2^ = 0.119; *F*(4,31) = 2.183; *p* = 0.094). Enjoyment was not significantly associated with any physiological outcome. These findings indicate that higher perceived exertion was the primary variable associated with elevated cardiovascular strain during moderate-intensity exercise sessions.

These findings have practical implications for exercise prescription in T1DM populations. When sophisticated monitoring tools are unavailable, assessing perceived exertion after moderate-intensity training sessions may assist exercise professionals in gauging cardiovascular strain and adjusting intensity accordingly. Additionally, monitoring enjoyment—although not independently associated with acute physiological strain—may inform strategies to preserve positive affective experience and support long-term adherence. However, neither RPE nor enjoyment should be relied upon to predict acute glycemic responses, which remain multifactorial and require direct blood glucose monitoring for safety. Given the exploratory nature of these findings and the small sample size, replication in larger, more diverse samples is needed before these results can inform clinical guidelines or exercise recommendations for adults with T1DM.

Future research should examine whether RPE is associated with blood glucose responses under more variable conditions, including higher-intensity protocols, longer-duration sessions, and less standardized insulin and carbohydrate management strategies. Additionally, investigation of whether enjoyment may emerge as a predictor of cardiovascular strain under more variable or higher-intensity exercise conditions is warranted.

## Figures and Tables

**Table 1:** Baseline characteristics of participants (*n* = 12).

Variables	Baseline Values *
Age, years	29.8 (7.8)
Sex male/female	7 (58.3)/5 (41.7)
Type 1 diabetes mellitus duration, years	11.9 (5.2)
Total body mass, kg	70.9 (17.6)
Height, m	1.67 (0.09)
Body mass index, kg·m^−2^	25.2 (4.6)
Σ Skinfolds, mm	215.8 (68.5)
Glycated hemoglobin—HbA1c, %	7.7 (1.6)
High-density lipoprotein (HDL) cholesterol, mg·dL^−1^	45.3 (7.8)
Low-density lipoprotein (LDL) cholesterol, mg·dL^−1^	119.5 (24.4)
Very-low-density lipoprotein (VLDL) cholesterol, mg·dL^−1^	27.2 (10.1)
Total cholesterol, mg·dL^−1^	193.6 (30.5)
Triglycerides, mg·dL^−1^	136.2 (50.7)
Hematocrit, %	44.4 (3.5)
Creatinine, mg·dL^−1^	1.16 (0.17)

* Data presented as mean (standard deviation) or *n* (%).

**Table 2:** Physiological and psychophysiological variables across three exercise sessions (*n* = 12).

Variables ^1^	Aerobic (*n* = 12)	Strength A (*n* = 12)	Strength B (*n* = 12)	*F*	*p*	*η* ^2^ *p*
HR rest, bpm	85.5 (9.3)	88.8 (8.2)	90.6 (8.1)	1.243	0.308	0.102
BG pre, mg·dL^−1^	204.6 (64.3)	208.0 (41.0)	182.4 (45.6)	1.117	0.329	0.092
BG immediately post, mg·dL^−1 †^	159.1 (68.1)	171.0 (47.2)	144.6 (53.8)	0.887	0.426	0.075
BG 20 min post, mg·dL^−1^	158.3 (59.4)	162.8 (56.6)	152.8 (58.7)	0.114	0.892	0.010
RPE, 0 to 10	4.0 (1.7)	6.0 (1.2)	6.7 (1.2)	12.368	<0.001 *	0.529
Enjoyment, 0 to 10	5.8 (2.6)	6.8 (2.3)	7.4 (1.6)	1.647	0.216	0.130
%HR, %	62.2 (9.7)	54.0 (10.7)	53.6 (14.8)	1.890	0.175	0.147

BG, blood glucose; HR, heart rate; RPE, rating of perceived exertion; %HR, percentage of heart rate reserve. ^1^ Data presented as mean (standard deviation). ^†^ For BG pre-exercise, Mauchly’s test indicated a violation of sphericity (*W* = 0.518, *p* = 0.037), and Greenhouse–Geisser-corrected values are reported for this variable. * Statistically significant (*p* < 0.05).

**Table 3:** Multiple linear regression models examining perceived exertion and enjoyment as predictors of cardiovascular and glycemic responses (*n* = 36).

Predictors ^1^	HR Rest B (95%CI)	BG Pre B (95%CI)	BG IP B (95%CI)	BG 20′ B (95%CI)	%HR B (95%CI)
RPE ^†^	−0.54 (−2.86, 1.77)	−3.85 (−17.38, 9.69)	−8.73 (−23.46, 6.01)	−6.66 (−21.94, 8.61)	3.05 (0.08, 6.02) * β = 0.44; *p* = 0.044
Enjoyment ^‡^	0.10 (−1.34, 1.55)	1.47 (−6.98, 9.92)	2.87 (−6.33, 12.07)	0.88 (−8.66, 10.41)	−0.33 (−2.18, 1.52) *β* = −0.06; *p* = 0.719
Adjusted *R*^2^	−0.051	−0.057	−0.025	−0.094	0.119
*F*	0.575	0.531	0.783	0.249	2.183

B, unstandardized regression coefficient; 95%CI, 95% confidence interval; BG, blood glucose; IP, immediately post-exercise; %HR, percentage of heart rate reserve. ^1^ Standardized β and *p*-values for the %HR model are reported both in the text and directly within table cells. Negative adjusted *R*^2^ values indicate that the model explains less variance than a model with no predictors, consistent with the near-zero *F*-statistics. Multiple linear regression with exercise session type (reference: STB) included as covariate. *n* = 36 (12 participants × 3 sessions). ^†^ RPE assessed using the Borg 6–20 scale (converted to 0–10) for aerobic sessions and the OMNI-RES 0–10 scale for resistance sessions. ^‡^ Enjoyment measured on a 0–10 visual analogue scale. * Statistically significant (*p* < 0.05).

## Data Availability

The datasets generated and/or analyzed during the current study are available from the corresponding author on reasonable request.

## Abbreviations

The following abbreviations are used in this manuscript:

%HR	Percentage of heart rate reserve
%HRmax	Percentage of maximum heart rate
1RM	One-repetition maximum
95%CI	95% confidence interval
AE	Aerobic exercise session
ANOVA	Analysis of variance
B	Unstandardized regression coefficient
BG	Blood glucose
BMI	Body mass index
CI	Confidence interval
HbA1c	Glycated hemoglobin
HDL	High-density lipoprotein
HR	Heart rate
IAE	Immediately after exercise
IDDM	Insulin-dependent diabetes mellitus
LDL	Low-density lipoprotein
OMNI-RES	OMNI Resistance Exercise Scale
RPE	Rating of perceived exertion
SD	Standard deviation
SE	Standard error
STA	Strength training session A
STB	Strength training session B
T1DM	Type 1 diabetes mellitus
UPE	University of Pernambuco
VIF	Variance inflation factor
VLDL	Very low-density lipoprotein
VO_2_max	Maximal oxygen uptake
β	Standardized regression coefficient
